# Supplementary material for: Asynchronous magnetic resonance elastography: Shear wave speed reconstruction using noise correlation of incoherent waves
Source: Magn Reson Med. 2022 Oct 27;89(3):990–1001. doi: 10.1002/mrm.29502 (PMC9792433; doi:10.1002/mrm.29502)
Supplement: Supplementary file 1 — DATA S1 MATLAB code used to generate the numerical simulations of Figure 1 [file MRM-89-990-s002.zip › k-Wave/helpfiles/example_ivp_heterogeneous_medium.html]

Heterogeneous Propagation Medium Example (k-Wave)


# Heterogeneous Propagation Medium Example

This example provides a simple demonstration of using k-Wave for the simulation and detection of the pressure field generated by an initial pressure distribution within a two-dimensional heterogeneous propagation medium. It builds on the Homogeneous Propagation Medium Example.

- Open the file in the MATLAB Editor
- Run the file in MATLAB

## Contents

- Defining a heterogeneous medium
- Running the simulation

## Defining a heterogeneous medium

For a homogeneous propagation medium, the sound speed is specified as a single scalar value in SI units. If the propagation medium is heterogeneous, `medium.sound_speed` and `medium.density` are instead given as matrices with the same dimensions as the computational grid (i.e., with `Nx` rows and `Ny` columns). These matrices can be created in several fashions, including explicitly (as given below), from external image maps, or using spatial or volume data from other simulations or experimental imaging modalities.

```
% define the properties of the propagation medium
medium.sound_speed = 1500 * ones(Nx, Ny);   % [m/s]
medium.sound_speed(1:Nx/2, :) = 1800;       % [m/s]
medium.density = 1000 * ones(Nx, Ny);       % [kg/m^3]
medium.density(:, Ny/4:Ny) = 1200;          % [kg/m^3]
```

## Running the simulation

The computation is again invoked by calling `kspaceFirstOrder2D` with the inputs defined above. Rather than manually producing a plot of the initial pressure distribution, sensor mask, and medium properties, it is also possible to use the inbuilt plotting functionality of `kspaceFirstOrder2D` by setting the optional input `'PlotLayout'` to `true`. This produces a plot of the simulation inputs as shown below. Optional inputs for k-Wave functions are given after the main inputs as `'string'`, `value` pairs. The `'string'` identifies the optional input parameter that is being modified, and the `value` is the user setting for this parameter. To remove the PML from the display, the optional input `'PlotPML'` is also set to `false`.

```
% run the simulation with optional inputs for plotting the simulation
% layout in addition to removing the PML from the display
sensor_data = kspaceFirstOrder2D(kgrid, medium, source, sensor, ...
    'PlotLayout', true, 'PlotPML', false);
```

A plot of the recorded time series is given below. The shape of the main wave-front has been perturbed compared to the homogeneous example, and weak reflections from the heterogeneous interfaces can also be seen.
